# *In Vivo* and *In Vitro* Characterization of the Immune Stimulating Activity of the Neisserial Porin PorB

**DOI:** 10.1371/journal.pone.0082171

**Published:** 2013-12-11

**Authors:** Andrew Platt, Heather MacLeod, Paola Massari, Xiuping Liu, Lee Wetzler

**Affiliations:** 1 Department of Microbiology, Boston University School of Medicine, Boston, Massachusetts, United States of America; 2 Section of Infectious Diseases, Department of Medicine, Boston Medical Center, Boston, Massachusetts, United States of America; International Center for Genetic Engineering and Biotechnology, India

## Abstract

Vaccines play a vital role in modern medicine. The development of novel vaccines for emerging and resistant pathogens has been aided in recent years by the use of novel adjuvants in subunit vaccines. A deeper understanding of the molecular pathways behind adjuvanticity is required to better select immunostimulatory molecules for use in individual vaccines. To this end, we have undertaken a study of the essential signaling pathways involved in the innate and adaptive immune responses to the *Neisseria meningitidis* outer membrane protein Porin B (PorB). We have previously demonstrated that PorB is an agonist of Toll-Like Receptor 2 (TLR2) and acts as an adjuvant in vaccines for protein, carbohydrate and lipopolysaccharide antigens using murine models. Here we demonstrate NFκB translocation following stimulation with PorB only occurs in the presence of TLR2. IL-6 and TNF-α secretion was shown to be MAPK dependent. Surface expression of activation markers on macrophages, including CD40, CD69, and CD86, was increased following PorB stimulation *in vitro*. Interestingly, some upregulation of CD54 and CD69 was still observed in macrophages obtained from TLR2 KO mice, indicating a possible non-TLR2 mediated activation pathway induced by PorB. In a murine vaccination model, using ovalbumin as the antigen and PorB as the adjuvant, a decreased antigen-specific IgG production was observed in TLR2 KO mice; adjuvant-dependent increased IgG production was entirely ablated in MyD88 KO mice. These observations demonstrate the importance of the above pathways to the adjuvant activity of PorB. The potential TLR2 independent effect is currently being explored.

## Introduction

Vaccines against infectious diseases have been one of modern medicines greatest tools in the last century [1]. Many of those pathogens that remain a risk in the 21^st^ century, however, have been stubbornly resistant to classical strategies of vaccine design [2-4]. Pathogens such as the *Plasmodium* *sp.*, HIV, mycobacterium and emerging pathogens present new obstacles to the development of effective immunotherapies [5].

The “dirty little secret” [6] of vaccines has long been trace contamination with Pathogen Associated Molecular Patterns (PAMPs): ligands for a wide range of extra- and intra-cellular Pattern Recognition Receptors (PRRs) [7]. The innate immune system has evolved multiple classes of PRRs, including the well known Toll-Like Receptors (TLRs) [8] and NOD-Like Receptors (NLRs)[9]. Ligation of these receptors by PAMPS activates cells of the innate immune system, which in turn can promote a stronger response by cells of the adaptive immune system[10-12]. Pathogen-derived molecules have therefore come under intense scrutiny as potential adjuvants for vaccines, especially subunit vaccines where the antigen itself is not highly immunogenic[13,14]. Extensive evidence exists of immune stimulation through TLR signaling demonstrating increased chemokine and cytokine production, up-regulation of co-stimulatory molecules and cell proliferation [15,16]. Common members of signaling networks, especially MyD88 [17-19], have also been observed to play a critical function in responses to PAMPs [20,21]. Currently, adjuvant selection for vaccines is determined primarily through extensive and costly empirical clinical testing of multiple adjuvant systems [22]. From a rational vaccine design standpoint, it should be possible to pick and choose among the vast array of available PAMPs for specific vaccine adjuvants each tailored to a desired outcome [7,23]. For this to be possible, however, a more detailed knowledge of the molecular mechanisms behind each ligand, and the skewing of the immune response generated by the presence of those ligands, is required. To this end, we set out to characterize, in more detail, the pathways activated by one particular TLR2 ligand, the outer membrane protein (OMP) Porin B (PorB) from *Neisseria meningitidis* [24].

Our laboratory has focused on investigating PorB and its interactions with the innate immune system. We have identified PorB as an agonist of TLR2/TLR1 heterodimers [25,26] and reported its ability to act as an adjuvant when used in conjunction with a wide array of antigens [27,28]. As a member of family of gram negative porins, PorB forms a trimeric β-barrel structure on the outer membrane of the bacteria, and serves as a pore for ion exchange [24,29]. In addition to identifying PorB as a TLR2/1 agonist, we have also made initial characterizations of the innate and adaptive response to the adjuvant *in vitro*; PorB increases surface expression of MHCII and CD86 on murine DCs and B cells and stimulates the release of Interleukin-6 (IL-6) and Tumor Necrosis Factor α (TNF-α) [30-33], both important signaling molecules in the inflammatory response. We have also studied Mitogen Activated Protein Kinase (MAPK) activation in response to PorB in B cells, focusing on the Erk1/2 pathway [34,35]. We chose to study select cell surface molecules on Antigen Presenting Cells (APCs) based on their role in the immune response or their known functions as markers of immune cell activation. Demonstrating that these effects are TLR2 dependent is, therefore, of interest in confirming the molecular mechanisms underlying the adjuvanticity of PorB. CD40 and CD86 are both involved in T cell stimulation by activated APCs [36-38]. An adhesion molecule, CD54 is involved in APC motility [39,40]. CD14 and CD69 were chosen as general markers of APC activation [41].

In the present study, we confirm the TLR2-dependent ability of PorB to induce secretion of inflammatory mediators in vitro and show evidence of MAPK signaling pathway induction We additionally report that PorB requires TLR2 to induce expression of multiple co-stimulatory molecules on Antigen Presenting Cells (APCs), and maximal antigen-specific IgG production when used as a vaccine adjuvant in the murine model. We additionally demonstrate that TLR2 is required for PorB to induce translocation of Nuclear Factor κB (NFκB) from the cytoplasm to the nucleus. Our results further clarify the complex molecular mechanisms behind the adjuvant activity of PorB and outline a more comprehensive picture of its activity on both the innate and adaptive immune system. These data also raise new questions about unexpected TLR2 independent activities of PorB, and present interesting topics for further exploration.

## Materials and Methods

### Mice

Six week old female wild type C57BL/6 mice were obtained from Jackson Laboratories (Bar Harbor, ME). MyD88 and TLR2 knockout mice [42] [43] (on the C57BL/6 genetic background) were a gift from Dr. S. Akiria (Research Institute for Microbial Diseases, Osaka University, Osaka, Japan). All mice were maintained within the Laboratory Animal Science Center (LASC) at Boston University School of Medicine under specific pathogen free (SPF) conditions. The Boston University Institutional Animal Care and Use Committee approved all research on animal models (permit number 14155). Care was taken to minimize animal pain and suffering.

### Vaccinations and serum collection

C57BL/6, TLR2 KO and MyD88 KO mice were vaccinated subcutaneously as described previously [30]. Vaccine formulations used 10 ug of lyophilized chicken egg Ovalbumin (Ova) with or without 10 µg PorB in 100 µL PBS per mouse. Control mice were given sham vaccines containing only 100 µl PBS. 3 doses were given on days 0, 14 and 28, blood was obtained from the tail vein on days -1, 13, and 27. On day 42, mice were humanely euthanized and terminal bleeds were obtained via cardiac puncture. Sera were frozen at -80 °C until use. Ova was derived from chicken egg whites by freeze-drying followed by lyophilization and resuspension of the total protein in sterile PBS. 

### PorB Purification

PorB was purified from *N. meninigitidis strain* H44/76 Δ-1/4 [44] as described previously [24]. by protein extraction and column chromatography. For use in vaccinations or cell stimulations, PorB was formed into protein micelles, termed proteosomes, as previously used and described [45].

### Generation of bone marrow derived macrophages (BMDM)

BMDM were generated from the femurs and tibias of C57BL/6, TLR2 KO and MyD88 KO mice [46]. Following the removal of muscle tissue, marrow was flushed from the bones with RPMI 1640 (Gibco, Life Technologies, Carlsbad, CA, USA). Single cell suspensions were generated by disruption using a 25G needle and passage through a 70 µm nylon mesh (ThermoFisher Scientific). Erythrocytes were lysed with NH_4_Cl, and the remaining cells pelleted, then plated in RPMI 1640 supplemented with 10% FBS (Gibco), 100 U/ml penicillin (Sigma-Aldrich, St. Louis, MO, USA), 100 µg/ml streptomycin (Sigma-Aldrich) and 20% 0.22 µm-filtered L929 (a M-CSF secreting cell line) conditioned media. Cells were plated in 10 cm bacterial plastic (ThermoFischer) plates. Media was changed every 3 days, washing the plates to purify macrophage progenitor cells by adherence to the plastic. Before experiments cells were removed from the plates by washing with trypsin and EDTA (Gibco) then plated at the appropriate density.

### Antibody and chemokines assays

Mouse sera were assayed for antigen-specific immunoglobulins by enzyme-linked immunosorbent assay (ELISA) as previously described [30]. Briefly, plate wells were coated with Ova (5 µg/mL) and incubated overnight at 4°C. Sera were sequentially diluted starting at 1:50 and added to the previously coated wells, and incubated at 37°C. Alkaline phosphotase-conjugated anti-mouse IgG, or anti-subtype IgG were added. After washing, the color was developed with one-step p-nitrophenyl phosphate (Pierce, Rockford, IL) and the optical density (OD) at 405 nm was measured on an ELx800 reader (Bio-Tek Instruments, Inc., Winooski, VT). Colorimetric values were converted to nanograms/milliliter, according to standard curves generated for total IgG; IgG subtypes were reported as the optical density (OD) of a 1:50 dilution of serum in PBS. Levels of IL-6 and TNF-α were measured in supernatants of BMDM cultures using ELISA kits (R&D Systems, Minneapolis, MN, USA and EBioscience, San Diego, CA, USA respectively) according to the manufacturer’s instructions.

### Staining for NFκB Translocation

C57BL/6 BMDMs were plated in Lab-TekII chamber slides (Nalge Nunc, Naperville, IL, USA) at 5 x 10^5^ cells/well. 48 hours later, cells were stimulated with the indicated ligand for 2 hours. Cells were washed and fixed with 4% paraformaldehyde for 30 minutes, then permeabilized with 0.2% Triton X-100 (ThermoFisher Scientific) in PBS. 200 µl Rabbit anti-mouse NFκB (Rockland Inc., Gilberstville, PA, USA) was used at 450 µg/ml, followed by 200 µl of 5 µg/mL goat anti-rabbit IgG Texas Red (Rockland Inc.). Counterstaining was done with 200 µl of a 150 ng/ml DAPI (Molecular Probes, Life Technologies, Carlsbad, CA, USA) solution. Cover slips (Corning, Corning, NY, USA) were placed on the washed slides and fluorescent microscopy was performed using a Nikon Eclipse (Nikon Instruments, Surrey, UK) and analyzed with Spot Basic 3.5.7 software (Diagnostic Instruments, Sterling Heights, MI, USA).

### BMDM Stimulation and IL-1β measurement

C57BL/6 BMDMs were plated in 24-well plates (Corning) at 5 x 10^5^ cells/well. 24 hours later, selected wells were stimulated in triplicate with one of the following TLR ligands: *E. coli* LPS double extracted in phenol chloroform to remove lipoprotein contamination, (100 ng/ml) (Sigma-Aldrich), Pam3CSK4 (100 ng/ml) (Invivogen, San Diego, CA, USA) or *N. meningitidis* porin PorB (10 ug/ml). 5 hours after stimulation, selected wells were stimulated with 5 mM ATP for 30 minutes. After 30 minutes, all supernatants were harvested and analyzed with the mouse IL-1β/IL-1F2 ELISA kit (R&D Systems). Briefly, Immulon 2 HB plates (ThermoFisher) were coated overnight with 4 ug/ml of rat anti-mouse IL-1β. Plates were washed and wells were blocked with PBS for 1 hour. Plates were washed and known concentrations of recombinant IL-1β or 1:2 dilutions of supernatant were added to the coated wells for 2 hours. Plates were washed and 2.5 ug/ml biotinylated goat anti-mouse IL-1β was added to the wells. Plates were washed and streptavidin-HRP was added to the wells. After washing the plates, a 1:1 mixture of H_2_O_2_ and TMB was added as a substrate for 20 minutes. Absorbencies were read at 450 nm. IL-1β concentrations in unknown samples were quantified using the standard curve.

### Cytokine production and MAPK inhibition

C57BL/6 BMDMs were plated as described above in 24-well plates (Corning) at 5 x 10^5^ cells/well. Cells were stimulated for 14 hours as described above with or without the presence of an inhibitor [35]. Supernatants were removed and stored at -20°C until analysis. Cytokine ELISAs for IL-6 and TNFα were performed using kits (BD OptEIA, San Jose, CA, USA) according to the manufacturer’s instructions. The following MAPK pathway inhibitors were used: UO126, an inhibitor of Erk1/2 activation by inhibiting MEK1/2 (Upstate, Lake Placid, NY, USA), SB203580, a direct inhibitor of p38 (Upstate), and SP600125, a direct inhibitor of Jnk (A.G. Scientific Inc., San Diego, CA, USA) [47]. Inhibitors were solublized in DMSO; vehicle controls were done using the highest concentration of DMSO used, alone.

### Stimulation and measurement of TLR expression

CD57B/L6 and TLR2 KO BMDMs were plated as above in 12-well plates (Corning) . Cells were stimulated for 24 hours with 200 ng/ml neisserial lipooligosaccharide (LOS; a gift from M. Apicella, University of Iowa, Iowa City, IA), Pam3CSK4 (100 ng/ml) or *N. meningitidis* PorB (10 ug/ml). Cells were harvested by gentle scraping, washed, incubated with FITC-labeled anti-TLR2 or anti-TLR4, then washed again and examined by flow cytometric analysis.

### Flow Cytometry

Flow cytometry [48] was conducted on BD FACScan and BD LSRII flow cytometers (Beckton Dickinson Biosciences, San Jose, CA). In brief, cells were harvested with gentle scraping washed with cold FACS buffer (0.2% BSA + 0.01% sodium azide in PBS) and stained for 30 minutes on ice in 150 µl of cold buffer containing 0.5 µg of the indicated fluorescent-labeled antibodies: FITC or PE rat IgG2a, FITC anti-CD69, FITC anti-IA^b^, FITC anti-CD14, FITC anti-CD54, FITC or PE anti-CD40, FITC, FITC or PE anti-CD86, FITC anti-TLR2, and FITC anti-TLR4 (Beckton Dickinson Biosciences, San Jose, CA and Caltag, Burlingame, CA). Forward and side scatter gates were used to identify live cells.

## Results

### MAPK is required for PorB-driven induction of pro-inflammatory cytokines

**Figure 1:**
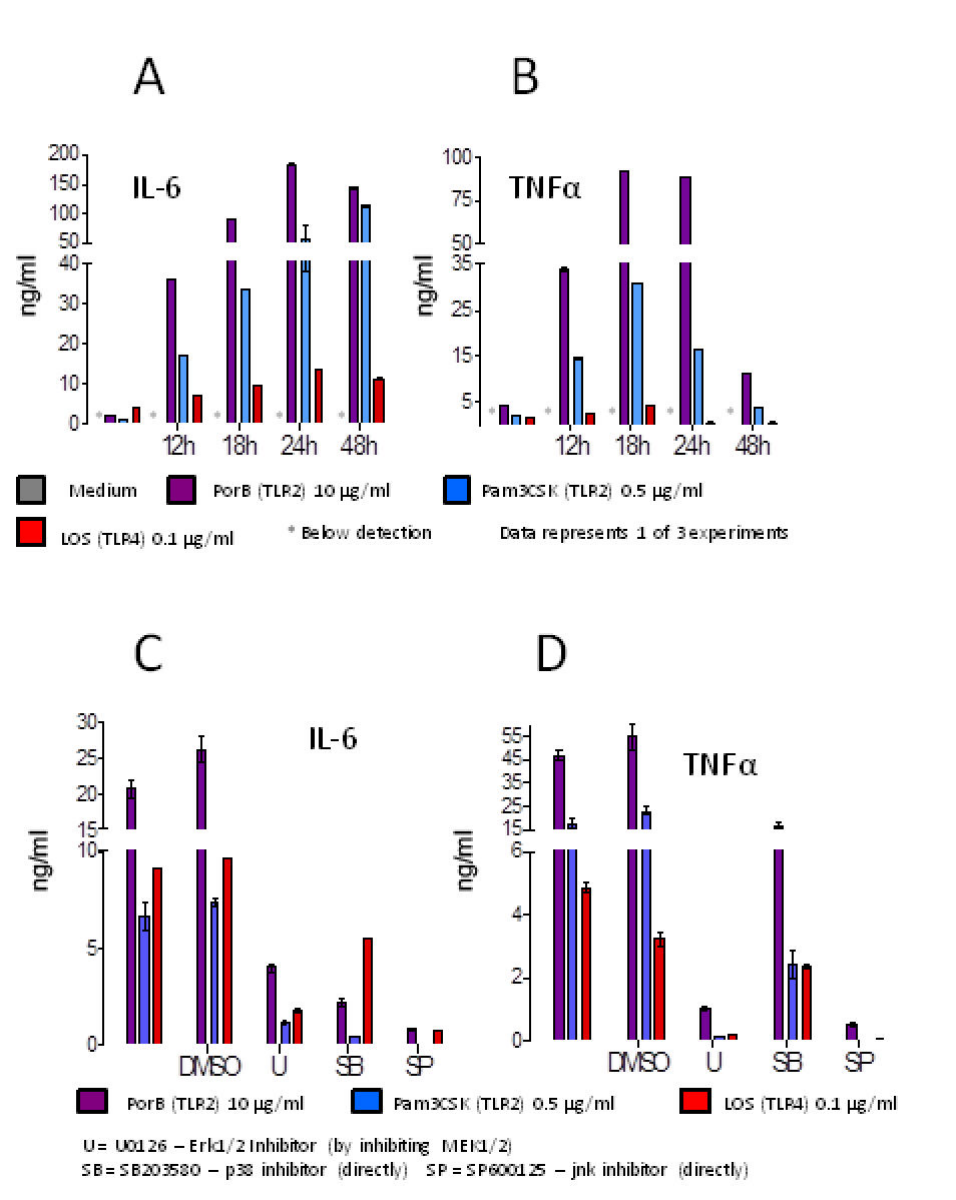
PorB induction of pro-inflammatory cytokines in macrophages is MAPK dependent. (a,b) PorB potently induces IL-6 and TNFα. Supernatants were collected from WT BMDM stimulated with PorB, Pam3CSK4 or *N. meningitidis* LOS at multiple time points. At 12, 24 and 48 hours post-stimulation levels IL-6 (a) of TNFα (b) were significantly higher than unstimulated cells. (c,d) Induction of inflammatory cytokines by PorB is inhibited by blocking MAPK. PorB, Pam3CSK4 or LOS were added to cultures of WT mouse BMDM. Cells were inhibited with the carrier DMSO, or MAPK pathway inhibitors, and production of inflammatory cytokines was measured. All inhibitors tested showed significant reductions in the stimulatory activity of PorB on the production of IL-6 (c) and/or TNFα (d). U: UO126, an inhibitor of Erk1/2 by inhibiting MEK1/2. SB: SB203580, a direct inhibitor of p38. SP: SP600125, a direct inhibitor of jnk. Data represents one of three experiments.

Secretion of cytokines by innate immune cells is a central mechanism for linking the innate and adaptive arms of the immune system [4,49,50]. Having previously shown that PorB initiates signaling by binding to TLR2 [25], we sought to further elucidate this signaling pathway. To demonstrate that PorB stimulated the release of pro-inflammatory cytokines, we measured the release of IL-6 and TNFα by C57BL/6 BMDMs in response to PorB. Pam_3_CSK_4_ and *N. meningitidis* lipoligosacharide (LOS) were used as TLR2 and TLR4 positive controls, respectively. At 12, 24 and 48 hours after stimulation both IL-6 and TNFα were present in significantly higher concentrations in the supernatants of wells stimulated with PorB compared to media alone as measured by ELISA (Figure **1a,b**). Cytokine concentrations in unstimulated wells were below the limit of detection. PorB-induced IL-6 concentrations in the supernatant peaked at 24-48 hours post stimulation, while TNFα concentrations peaked at 18-24 hours after addition of PorB. At 10 µg/ml, PorB induced IL-6 and TNFα concentrations comparable to stimulation by 0.5 µg/ml PAM_3_CSk_4_, and significantly higher than 100 ng/ml *N. meningitidis* LOS.

We next determined if the secretion of these cytokines was dependent on MAPK signaling by blocking signaling of pathway intermediates. We pretreated mouse BMDMs with MAPK pathway inhibitors as indicated, then stimulated the cells with the same TLR ligands as before. UO126 was used for indirect inhibition of Erk1/2 by inhibiting MEK1/2, SB203580 is a direct inhibitor of p38, and SP600125 is a direct inhibitor of jnk. All three of the inhibitors decreased IL-6 (Figure **1c**) and TNFα (Figure **1d**) secretion by the BMDMs in response to PorB. MAPK inhibition by all three inhibitors was additionally observed to decrease cytokine release in response to both PAM3CSK4 and LOS. No significant effect was observed in response to treatment with DMSO. Direct inhibition of p38 was observed to have a partial effect on TNFα release when compared to inhibition of either Erk1/2 or jnk, but still resulted in a 3-fold reduction in the concentration of TNFα in the supernatant when compared to the empty vehicle.

#### Cell surface markers are upregulated on BMDMs following stimulation with PorB

**Figure 2:**
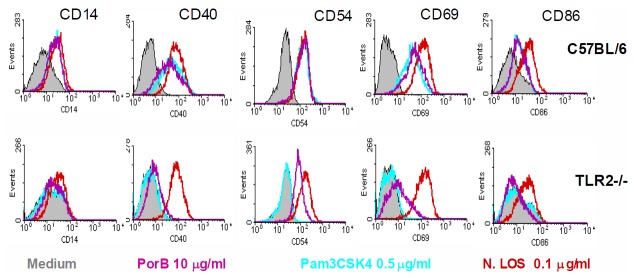
Upregulation of co-stimulatory cell-surface proteins by PorB is partially TLR2 dependent. WT and TLR2 KO BMDMs were stimulated in culture with PorB, Pam3CSK4 or LOS, then stained for markers of activation and examined by flow cytometry. In WT cells, PorB increased surface expression of CD14, CD40, CD54, CD69 and CD86 as compared to unstimulated cells. Knockout cells had decreased expression of CD14, CD40 and CD86, but still had higher expression of CD54 and CD69 when compared to basal levels. In the latter two cases, the TLR2 ligand Pam3CSK4 had no effect on TLR2 -/- cells. Histograms are from one representative sample of at least 3. Data represents one of two experiments.

Complementary to cytokine secretion, presentation of co-stimulatory cell surface proteins on APCs is both a measure of activation and a mechanism of communication between the innate and adaptive arms of the immune system [39,41]. We investigated proteins significant for T cell stimulation, (CD40, CD86) [36-38] and for APC motility (CD54) [39,40], as well as other markers of BMDM activation (CD14, and CD69) [41]. We have previously shown that PorB induces upregulation of CD86 and MHCII on APCs, but not CD80 [31]. C57BL/6 and TLR2 KO cells were stimulated with PorB, or Pam_3_CSK_4_ and LOS as positive controls for 24 hours, then stained for surface proteins and analyzed by flow cytometry. All five cell surface proteins were upregulated by PorB in WT cells. In TLR2 KO cells following PorB stimulation CD14, CD40 and CD86 were not upregulated relative to unstimulated cells. (Figure **2**) Unexpectedly, CD54 and CD69 were partially upregulated in TLR2 KO cells following stimulation with PorB. All five markers were not upregulated in TLR2 KO cells stimulated with Pam_3_CSK_4_. All five molecules were upregulated following stimulation with the TLR4 ligand LOS. LOS induced upregulation remained constant in TLR2 knockout cells. We also extended to macrophages previous results wherein nuclear translocation of NFκB was observed in B cells following stimulation with PorB [34]; as in our previous work translocation was not observed when cells derived from TLR2 KO mice were stimulated with PorB (data not shown).

**Figure 3:**
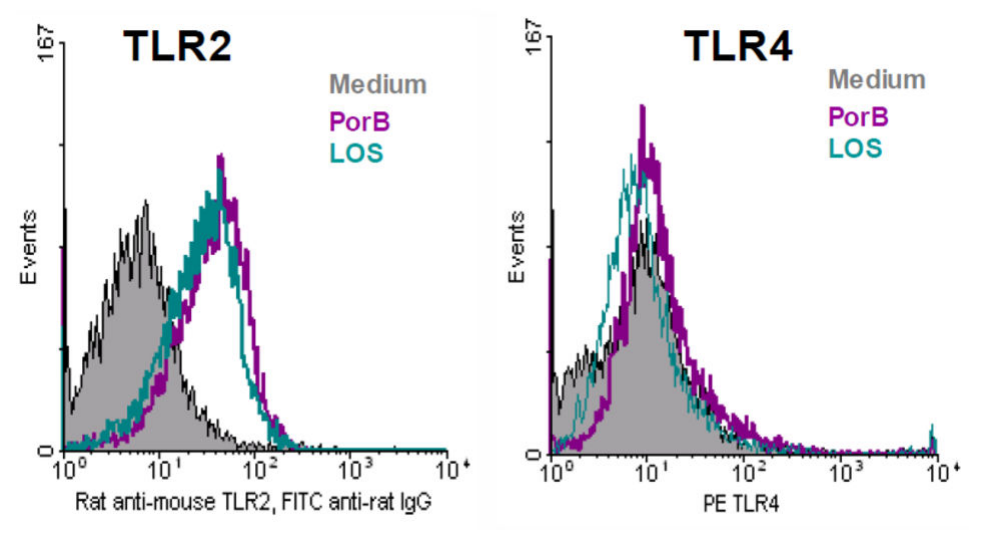
PorB increases surface expression of TLR2. C57Bl/6 mouse BMDM were stimulated with PorB or LOS. Stimulation lead to increased surface expression of TLR2 following stimulation with either TLR ligand when compared to media controls. Neither ligand, however, induced surface expression of TLR4. Histograms are from one representative sample of at least 3. Data represents one of two experiments.

To investigate the possibility of a positive feedback loop, we determined if there was increased surface expression of TLR2 and TLR4 on BMDMs following stimulation with PorB or LOS. Cells were stimulated *in vitro* for 24 hours, then assayed by flow cytometry. Stimulation with either PorB or LOS led to increased surface expression of TLR2 as compared to unstimulated controls; neither ligand increased surface expression of TLR4 (Figure **3**).

### PorB alone is not sufficient to activate the inflammasome

**Figure 4:**
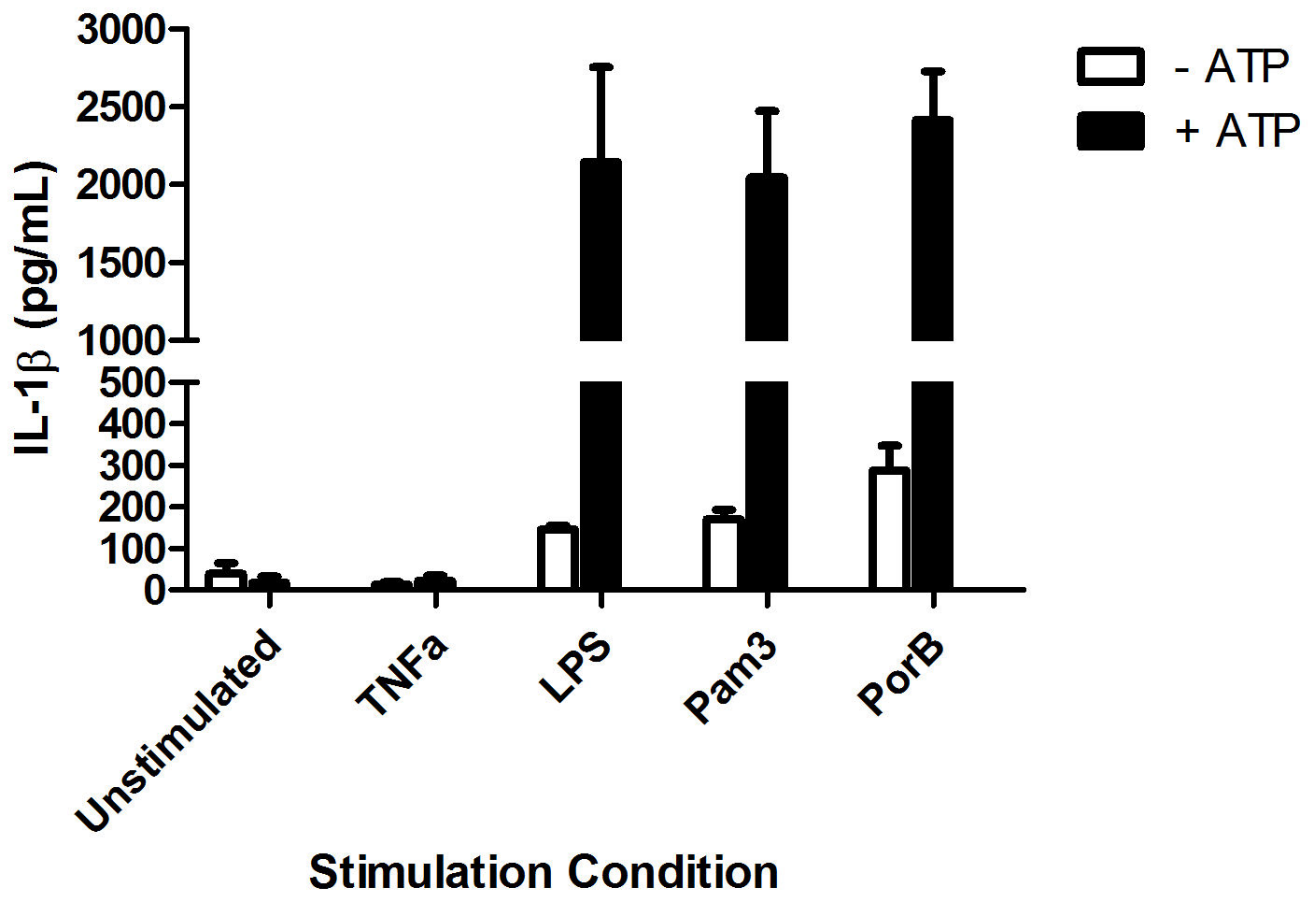
IL-1β induction by PorB requires ATP to activate the inflammasome. WT BMDMs were stimulated with PorB, LPS, TNFα, or Pam_3_CSK_4_ at the indicated concentrations for 5 hours. After 5 hours, ATP was added to half the cells for 30 minutes. Supernatants were collected and analyzed by IL-1β ELISA. IL-1β release was not observed for PorB, LPS or Pam_3_CSK_4_ in the absence of ATP. In the presence of ATP, significantly increased IL-1β release was observed for all three TLR ligands (p<0.05).

Activation of the inflammasome is a possible mechanism of action for adjuvants that is of significant interest [51,52]. We investigated if stimulation with PorB alone was sufficient to activate the inflamasome *in vitro*. Release of cleaved IL-1β requires activation of both signals for the inflamasome: increased pro-IL-1β synthesis and cleavage of pro-IL-1β by activated Caspase 1 [51]. C57BL/6 BMDMs were stimulated with PorB, LPS or Pam_3_CSK_4_. After 5 hours, exogenous ATP was added to half of the wells to trigger activation of Caspase 1 [53]. In the absence of ATP, none of the TLR ligands stimulated the release of IL-1β as measured by ELISA. However, increased mature IL-1β following ATP co-stimulation was observed for all of the TLR ligands tested including PorB (p<0.05) (Figure **4**).

### MyD88 and TLR2 are necessary for the maximal adaptive immune response to PorB

**Figure 5:**
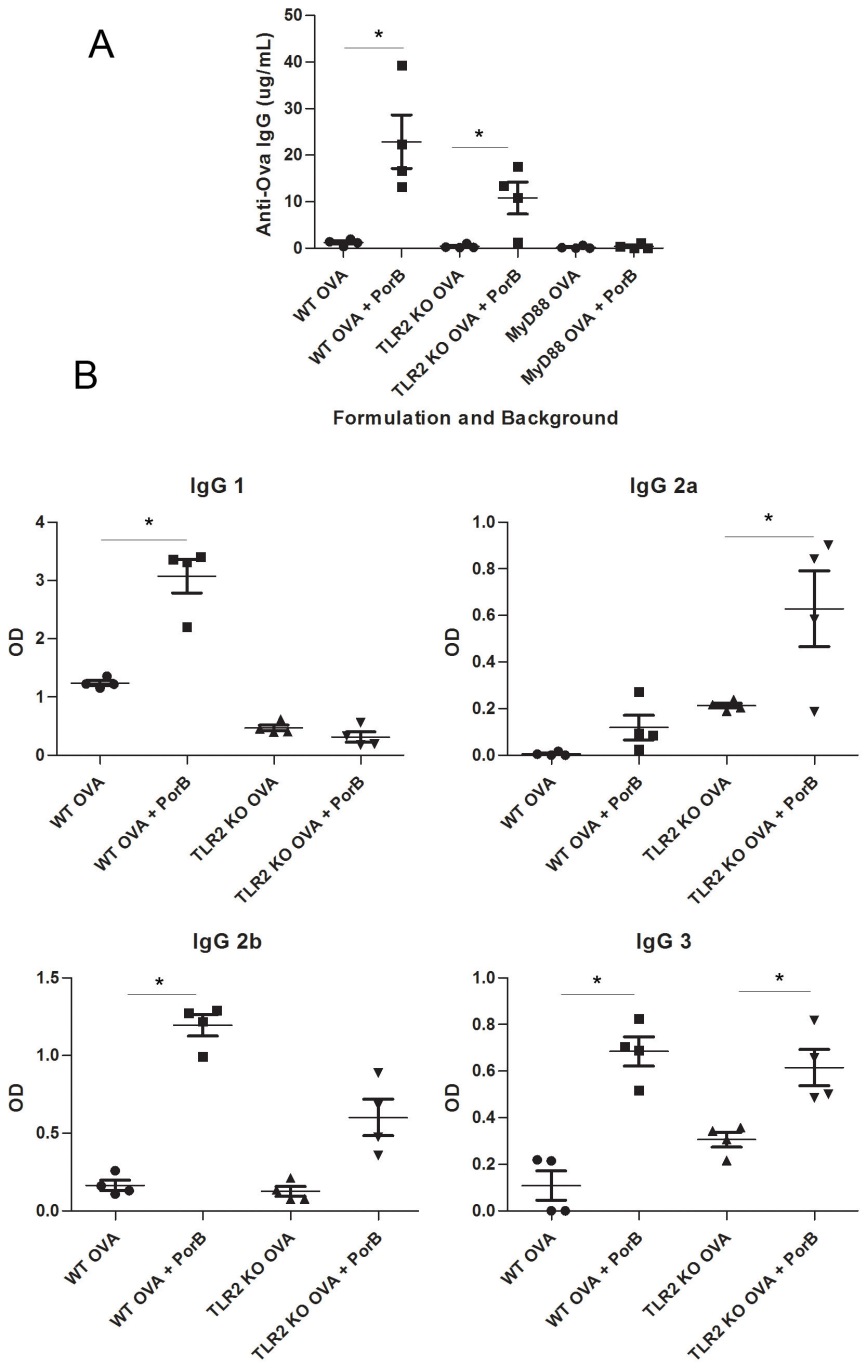
Increases in antigen-specific IgG by PorB is dependent on TLR2 and MyD88. (a) Concentration of IgG antibody to Ova in C57Bl/6 mice as measured by ELISA in serum on from day 42. WT C57Bl/6, TLR2 ^-/-^ and MyD88 ^-/-^ mice were vaccinated on days 0, 14 and 28 with Ova, Ova + PorB or sham (PBS). Vaccines that included PorB showed significantly less elevation in IgG over vaccines that did not include PorB in TLR2 ^-/-^ mice, and no detectable effect in MyD88 ^-/-^ mice. None of the mice had detectable antibodies to Ova prior to vaccination (data not shown). (b) OD of specific subtypes of anti-Ova IgG at a 1:50 dilution of serum as measured by ELISA. In WT mice, IgG1 and IgG2b were the dominant subtypes indicative of a Th2-type response. These in turn decreased in the TLR2 ^-/-^ mice, consistent with the total IgG data. Data represents one of two experiments. * p<0.05.

Previous work by our lab has show that PorB is a TLR2 agonist [25], and that it functions as an adjuvant in vaccines [27]. To determine whether TLR2 or its downstream adapter protein, MyD88, are required for the adjuvant activity of PorB, we vaccinated mice lacking each gene. TLR2 KO and MyD88 KO mice, in addition to WT C57BL/6 mice, were vaccinated with Ova or a combination of Ova and PorB. Vaccines were administered subcutaneously (s.c.) on days 0, 14, and 28. The level of the humoral immune response generated towards the antigen (Ova) was then deteremined. Blood was collected from the mice on day 42, and the serum assayed by ELISA for Ova-specific IgG. Consistent with previous work, WT mice had significantly higher concentrations of Ova-specific IgG when vaccinated with Ova + PorB as compared to Ova alone. TLR2 KO mice vaccinated with Ova + PorB had significantly lower concentrations of Ova-specific IgG than WT mice given the same vaccine, but higher levels of IgG than TLR2 KO mice vaccinated with Ova alone. (Figure **5a**). For all mice, anti-Ova IgG in pre-immune sera was below the limit of detection (data not shown). Given that PorB has been shown to be a TLR2 agonist in previous work, any adjuvant activity by PorB in the TLR2 KO mouse was not expected. We have ruled out contamination by endotoxin by Limulus Amoebacyte Assay and by nucleic acids by running samples of PorB on 2% agarose gels and staining with ethidium bromide (data not shown). Despite the slight retention in adjuvanticity of PorB in the TLR2 KO mice, the substantial decrease in IgG from the WT mouse model demonstrates the importance of TLR2 signaling to the adjuvant effects of PorB. In contrast, MyD88 KO mice vaccinated with Ova showed no increase in the level of Ova-specific IgG when PorB was added to the vaccine formulation.

We further investigated the dependence of the adjuvant activity of PorB on TLR2 to determine if it would be similar for the individual IgG subtypes. Subtyping IgG can additionally be used to characterize the immune response to PorB as skewing towards a Th1 or Th2 phenotype [54]. The relative amount of OVA specific IgG1, IgG2b, IgG2c and IgG3 was determined in sera obtained from vaccinated mice two weeks after the third immunization (Day 42). The OD at a single, fixed dilution of 1:50 was determined as quantitation of IgG subtypes could not be performed in the absence of suitable purified IgG subtype standards.. WT mice vaccinated with Ova + PorB demonstrated an immune response dominated by IgG1 and IgG2b (Figure **5b**). These subtypes, in turn, were significantly suppressed in the responses of TLR2 KO mice given the same vaccine. These data are consistent with induction of a Th2 type response [55], and demonstrate that both the induction of the response and its phenotype are dependent on TLR2-mediated processes. Interestingly, immunization of TLR2 KO mice resulted in a statistically significant (p<0.05) increase in IgG2c production as compared to WT mice, possibly indicating that the non-TLR2 mediated adjuvant activity of PorB may induce more of Th1 type response, as IgG2c induction is related to production of Th1 type cytokines (i.e. IL-12). However, it is unclear if the increase has a physiological relevance. 

## Discussion

The use of immune adjuvants, other than alum, in human vaccines is beginning to be explored [13,22,56]. These adjuvants are essential for designing vaccines that avoid the potential dangers of attenuated or inactivated pathogens, while still eliciting strong protective immune responses. Therefore, there is great interest in developing and characterizing new adjuvant candidates with novel function and mechanisms of action [57-59]. Research into vaccines adjuvants has generally focused on identifying candidate adjuvants and measuring their efficacy in model systems [16]. While broad trends have emerged in the classes of compounds that are effective adjuvants [60], including the TLR ligands [15,16], less work has been done to determine the specific molecular mechanisms by which individual adjuvants can be differentiated from one another. One of the aims of our research is to elucidate the mechanisms by which one specific TLR ligand can act as a vaccine adjuvant. The unique set of pathways affected by each adjuvant is not fully understood, especially for newer adjuvants such as PorB [25,28]. We set out to test a number of possible related pathways through which PorB may be stimulating the innate immune system as an adjuvant, and thus influencing the adaptive response to a vaccine antigen. Additionally, we sought confirmation that the activation of these pathways occurs *via* signaling through the known TLR2 agonist activity of PorB. 

In previous work, we have shown that the *N. meningitidis* OMP PorB acts as an adjuvant in vaccines, increasing the strength of adaptive immune responses to a range of antigens, including polysaccharides, proteins, and *F. tularensis* LPS [27,31]. We have also demonstrated that it is a ligand of TLR2/1 heterodimers [25], and requires MyD88 for efficient cell signaling [24,61]. Analysis of the TLR2 dependent effects of PorB are therefore essential in linking its adjuvant activity to known pathways of innate activation. Such a demonstration would establish a clear causal chain, validating our proposed mechanism of adjuvanticity.

In this report, we have shown that PorB acts through multiple downstream signaling pathways to stimulate the innate immune system and trigger signals that propagate through to the adaptive immune system. On the innate side, we have observed signaling through the NFκB and MAPK pathways, while seeing no evidence for signaling through the inflammasome and direct production of IL-1β. Furthermore, we see evidence for a positive feedback loop whereby agonism of TLR2 by PorB results in increased surface expression of the receptor. In communication with the adaptive arm of the immune system, ligation of TLR2 by PorB results in the surface expression of a number of co-stimulatory molecules on APCs and induces secretion of key cytokines IL-6 and TNFα. The majority of these effects are, as hypothesized, TLR2-dependent. This agrees well with the observation that the overall immune response as measured by the serum levels of antigen-specific IgG decrease significantly in the IgG knockout mice. Unexpectedly, however, we observed a few outcomes that did not fully return to baseline in the TLR2 knockout mouse, including expression of CD54 and CD69, and antigen-specific IgG. This would suggest that there may be a secondary, likely weaker, stimulus provided by PorB that is independent of the TLR2 pathways observed elsewhere in this report. Based on known characteristics of PorB, that it forms ion pores in the outer membrane of *N. meningitidis* and in our preparations aggregates into nanoscale proteosome structures [24,29,62], a number of hypothetical mechanisms suggest themselves. PorB may be creating pores on the cell surface, leading to ion flux across the plasma membrane and triggering activation of APCs [63]. The observed response may alternately be due to particulate stimulation by PorB proteosomes on APCs; inflammatory stimulation by particulates has been observed to be a MyD88-dependent process [64]. 

Previously we have demonstrated that murine DCs secrete the pro-inflamatory cytokines IL-6 and TNFα when stimulated by PorB [61]. Furthermore, connections between TLRs and the MAPK pathway have been extensively studied [65]. After confirming that both cytokines were produced in BMDMs as well when stimulated with PorB, we set out to demonstrate that the MAPK pathway was an essential mediator in the secretion of these cytokines in response to PorB stimulation. In line with our hypothesis, IL-6 and TNFα production, while high in C57Bl/6 BMDMs, was greatly ablated in the presence of MAPK pathway inhibitors. The use of UO126, SB203580, and SP600125 allowed us to confirm that each successive member of the MAPK pathway was essential to complete the signaling cascade leading to cytokine production. As in other experiments, a TLR2/1 agonist and a TLR4 agonist allowed us to control for on- and off-target receptor specificity of the signaling cascade. These results thus demonstrate that the secretion of cytokines by macrophages in response to stimulation with PorB occurs downstream of the MAPK signaling cascade. Significant evidence exists in the literature detailing NFκB-dependent processes downstream of TLR2 signaling events [16]. We sought to extend our previous demonstration of NFκB signaling in B cells [34] to macrophage cell lines in support of a hypothesis that the immune stimulatory effects of PorB cover the breadth of the immune system. As expected, TLR2 KO cells did not display nuclear translocation of NFκB, confirming the essential role of TLR2 agonism by PorB in this process. 

The role of the inflammasome and IL-1β in vaccine adjuvant activity is a controversial topic, especially in regards to the mechanism of action of alum [51,52]. In particular, while TLRs are known to increase transcription and translation of pro-IL-1β, activation of an NLR or AIM2 is still required to cleave pro-caspase-1 and begin the inflamasome cascade leading to the production of active IL-1β and IL-18 [9]. As *N. meningitidis* PorB is a pore-forming protein, and potassium efflux driven by exogenous ATP is sufficient to activate NLRP3 [66], we were interested to see if PorB alone would be sufficient to provide both signals to the inflamasome. This would provide a novel mechanism for a TLR-based adjuvant to stimulate the immune system and could possibly explain some of the TLR2-independent, MyD88-dependent effects we observed in other experiments. However, our findings demonstrated that while PorB was indeed capable of promoting IL-1β release when given in combination with exogenous ATP, PorB alone, like Pam_3_CSK_4_ and LPS, failed to elicit a strong response. The lack of IL-1β generated by treatment with PorB alone suggests an alternative explanation is needed for the weak adjuvant activity of PorB in TLR2 KO mice.

The ultimate goal for any vaccine adjuvant is to elicit a robust antigen-specific response by the adaptive immune system [4]. As the canonical targets of many adjuvants, including PorB, are elements of the innate immune system, this requires efficient communication between the two arms. To this end, we were interested in the expression of surface proteins on APCs in response to PorB. Previous research by our lab had identified MHCII and CD86 as upregulated following stimulus by PorB [61]. As additional targets, we chose proteins significant for T cell stimulation (CD40) [36], for APC motility (CD54) [40], and markers of BMDM activation (CD14, and CD69) [41]. We observed increases in all of these antigens following stimulation with PorB, suggesting that APCs thus exposed *in vivo* would be well prepared to activate CD4 T cells. This agrees with previous work by our lab showing significant increases in the CD4 T cell dependent process of class switch recombination driven by the inclusion of PorB in vaccine formulations [27], and confirmed by our observation of significantly increased antigen-specific IgG in C57Bl/6 mice vaccinated with Ova + PorB as compared to mice vaccinated with Ova alone. Surprisingly, CD54 and CD69 expression in TLR2 KO BMDMs stimulated with PorB were still slightly elevated when compared to unstimulated cells. Additionally, while TLR2 KO mice vaccinated with Ova + PorB had anti-Ova IgG levels significantly below those of WT mice given the same vaccine, they still had higher antibody levels than TLR2 KO mice vaccinated with Ova alone. While we do not as yet have a confirmed mechanism for this weaker adjuvant activity, it does appear to be MyD88-dependent, as MyD88 KO mice vaccinated with Ova + PorB and anti-Ova IgG levels comparable to those vaccinated with Ova alone. This is an area of exploration that we are currently pursuing.

In summary, we demonstrate that *Neisseria meningitidis* PorB acts in a TLR2 and MyD88 dependent manner when stimulating the innate immune system and increasing antigen-specific IgG production in the murine model. We have clarified the roles of MAPK and NFκB in the immune response to PorB, while raising question that may prove rewarding of future work.
